# Large variation in radiation dose for routine abdomen CT: reasons for excess and easy tips for reduction

**DOI:** 10.1007/s00330-023-10076-6

**Published:** 2023-09-21

**Authors:** Rebecca Smith-Bindman, Taewoon Kang, Philip W. Chu, Yifei Wang, Carly Stewart, Marco Das, Phuong-Anh Duong, Luisa Cervantes, Ramit Lamba, Ryan K. Lee, Fiona MacLeod, Nima Kasraie, Rebecca Neill, Pavlina Pike, Jodi Roehm, Sebastian Schindera, Robert Chung, Bradley N. Delman, Cécile R L P N Jeukens, L. Jay Starkey, Timothy P. Szczykutowicz

**Affiliations:** 1 Department of Epidemiology and Biostatistics, University of California San Francisco, 550 16Th Street, San Francisco, CA 94158 USA; 2 Department of Obstetrics, Gynecology and Reproductive Sciences, University of California, San Francisco, CA USA; 3 Philip R. Lee Institute for Health Policy Studies, University of California San Francisco, 490 Illinois Street, San Francisco, CA 94158 USA; 4 Department of Diagnostic and Interventional Radiology, Helios Hospital Duisburg, An Der Abtei 7-11, 47166 Duisburg, Germany; 5 Department of Radiology, New York University Langone, 6 Ohio Drive, Lake Success, NY 11042 USA; 6 Department of Radiology, Nicklaus Children’s Hospital, 3100 SW 62Nd Avenue, Miami, FL 33155 USA; 7 Department of Radiology, University of California Davis, 4860 Y Street, Suite 3100, Sacramento, CA 95817 USA; 8 Department of Radiology, Ground Floor, Einstein Healthcare Network, 5501 Old York Road, Levy Bldg, Philadelphia, PA 19141 USA; 9 Department of Radiology, John Radcliffe Hospital, Oxford University Hospitals NHS Foundation Trust, Oxford, OX3 9DU UK; 10 Department of Radiology, University of Texas Southwestern Medical Center, 5323 Harry Hines Blvd, Dallas, TX 75390 USA; 11 Department of Radiology and Imaging Sciences, Emory University, 1365 Clifton Road NE, Atlanta, GA 30322 USA; 12 Huntsville Hospital, 101 Sivley Rd SW, Huntsville, AL 35801 USA; 13 Navvis Healthcare, Missouri, USA; 14 Institute of Radiology, Kantonsspital Aarau AG, Tellstrasse 25, 5001 Aarau, Switzerland; 15 Department of Demography, University of California Berkeley, 310 Social Sciences Building, Berkeley, CA 94720-2120 USA; 16 Department of Diagnostic, Molecular and Interventional Radiology, Icahn School of Medicine at Mount Sinai, One Gustave L. Levy Place, New York, NY 10029-6574 USA; 17 Department of Radiology and Nuclear Medicine, Maastricht University Medical Centre+, P. Debyelaan 25 6229 HX, PO Box 5800, 6202 AZ Maastricht, The Netherlands; 18 Department of Radiology, St Luke’s International Hospital, 9-1 Akashicho, Tokyo 104-8560 Chuo City, Japan; 19 Departments of Radiology, Medical Physics, and Biomedical Engineering, University of Wisconsin Madison, Madison, WI USA

**Keywords:** Tomography, X-ray computed, Abdomen, Radiation dosage

## Abstract

**Objective:**

To characterize the use and impact of radiation dose reduction techniques in actual practice for routine abdomen CT.

**Methods:**

We retrospectively analyzed consecutive routine abdomen CT scans in adults from a large dose registry, contributed by 95 hospitals and imaging facilities. Grouping exams into deciles by, first, patient size, and second, size-adjusted dose length product (DLP), we summarized dose and technical parameters and estimated which parameters contributed most to between-protocols dose variation. Lastly, we modeled the total population dose if all protocols with mean size-adjusted DLP above 433 or 645 mGy-cm were reduced to these thresholds.

**Results:**

A total of 748,846 CTs were performed using 1033 unique protocols. When sorted by patient size, patients with larger abdominal diameters had increased dose and effective mAs (milliampere seconds), even after adjusting for patient size. When sorted by size-adjusted dose, patients in the highest versus the lowest decile in size-adjusted DLP received 6.4 times the average dose (1680 vs 265 mGy-cm) even though diameter was no different (312 vs 309 mm). Effective mAs was 2.1-fold higher, unadjusted CTDI_vol_ 2.9-fold, and phase 2.5-fold for patients in the highest versus lowest size-adjusted DLP decile. There was virtually no change in kV (kilovolt). Automatic exposure control was widely used to modulate mAs, whereas kV modulation was rare. Phase was the strongest driver of between-protocols variation. Broad adoption of optimized protocols could result in total population dose reductions of 18.6–40%.

**Conclusion:**

There are large variations in radiation doses for routine abdomen CT unrelated to patient size. Modification of kV and single-phase scanning could result in substantial dose reduction.

**Clinical relevance:**

Radiation dose-optimization techniques for routine abdomen CT are routinely under-utilized leading to higher doses than needed. Greater modification of technical parameters and number of phases could result in substantial reduction in radiation exposure to patients.

**Key Points:**

*• Based on an analysis of 748,846 routine abdomen CT scans in adults, radiation doses varied tremendously across patients of the same size and optimization techniques were routinely under-utilized.*

*• The difference in observed dose was due to variation in technical parameters and phase count. Automatic exposure control was commonly used to modify effective mAs, whereas kV was rarely adjusted for patient size. Routine abdomen CT should be performed using a single phase, yet multi-phase was common.*

*• kV modulation by patient size and restriction to a single phase for routine abdomen indications could result in substantial reduction in radiation doses using well-established dose optimization approaches.*

**Supplementary information:**

The online version contains supplementary material available at 10.1007/s00330-023-10076-6.

## Introduction

CT utilization has grown significantly in the last three decades, with an estimated 91 million scans performed in the United States in 2019 [1] and 90 million scans in the European Union in 2020 [2]. Concern over the corresponding increase in exposure to ionizing radiation has led to broad interest in radiation dose optimization and in avoiding unnecessary exams and non-indicated scan series [3–11]. Despite this attention, large variation in dose persists [9, 12–15].

Extensive work has been published on dose reduction approaches. There have been meaningful technological developments, such as automatic exposure control (AEC) and iterative reconstruction [16], as well as educational efforts to encourage modification of technical parameters, such as the use of lower tube potential (kilovoltage, kV) and size-specific protocols [17–20]. However, little is known to what extent radiology providers employ these techniques, or the impact on radiation dose when they are used. Understanding current practice could drive improvement.

Using observed CT data from a large radiation dose registry, we describe the frequency with which different technical parameters are used for routine abdomen CT and the impact on patient radiation dose when dose reduction strategies are or are not implemented.

## Methods

### Study population

Drawing from the University of California, San Francisco (UCSF) International CT Dose Registry (“registry” [13]), we retrospectively analyzed consecutive diagnostic CT scans performed from January 1, 2015 to October 21, 2020, in patients aged 18 years and older. The registry pools 100% of CT scans performed at imaging facilities from 27 healthcare organizations in 7 countries, all of which submitted data via Radimetrics© dose management software (Bayer HealthCare). The data include the four largest CT manufacturers (Table 1) [12, 13]. The UCSF Committee on Human Research approved this study with a waiver of informed consent. Collaborating institutions obtained local Institutional Review Board approval or relied on UCSF approval to contribute to the registry.

**Table 1:** Routine abdomen and pelvis CT scans included in this report

	*n*	%
Total number of scans	748,846	100.0
Sex		
Men	321,648	43.0
Women	426,840	57.0
Non-binary or unknown	358	0.0
Age		
18–19	9,169	1.2
20–29	67,695	9.0
30–39	89,195	11.9
40–49	110,103	14.7
50–59	138,768	18.5
60–69	142,551	19.0
70–79	111,508	14.9
80–89	63,881	8.5
90–99	15,976	2.1
Manufacturer		
Canon	58,815	7.9
General Electric	298,316	39.8
Philips	146,937	19.6
Siemens	244,778	32.7
Country		
Germany	6,565	0.9
Israel	33,941	4.5
Japan	8,732	1.2
Netherlands	13,829	1.8
Switzerland	8,963	1.2
UK	11,515	1.5
USA	665,301	88.8

For each CT scan, patient sex and age, effective patient diameter, CT category (reflecting the body region and image quality requirements [21]), scanner manufacturer and model, and the radiation dose metrics and technical parameters of the scan were extracted from the registry [12, 13, 22]. These analyses focus on scans obtained for routine abdomen CT, determined using information contained in the Digital Imaging and Communications in Medicine (DICOM) headers. Abdomen scans obtained for low-dose indications (e.g., suspected renal stones) or high-dose indications (e.g., cancer surveillance or assessment of acute intra-abdominal bleeding) are not included in this manuscript. A full list of indications included/excluded in routine abdomen CT, and the validation of the framework for assigning CT scans to categories demonstrating 90% accuracy compared with expert review, has been previously published [21]. Routine abdomen is one of the most common reasons patients undergo CT imaging, accounting for approximately 24% of all CT scans [21]. This category most closely aligns with the EUCLID abdomen, appendicitis category [12].

### Radiation dose variables

Radiation dose metrics included the machine-reported volume computed tomography dose index (CTDI_vol_) and dose length product (DLP), and effective dose, which is calculated by Radimetrics and reflects future cancer risk. Radiation dose varies non-linearly by patient size, so that in general larger patients require asymmetrically higher doses than smaller patients to achieve sufficient image quality. To minimize the impact of patient size on variation across practices, we calculated size-adjusted CTDI_vol_ and size-adjusted DLP by normalizing these metrics using the log-linear mixed regression between them and effective patient diameter (patient diameter, defined as the average diameter measured by Radimetrics on axial or scout images); the facility at which the scan was performed was included as a random effect. The following equations were used where the population median abdominal diameter = 303 mm:

$${\mathrm{ADJCTDI}}_{\mathrm{vol}}={\mathrm{CTDI}}_{\mathrm{vol}}\times\exp\left(-\left(\mathrm{EFFECTIVE}\;\mathrm{PATIENT}\;\mathrm{DIAMETER}-303\right)\times0.007682\right)$$

$$\mathrm{ADJDLP}=\mathrm{DLP}\;\times\;\exp(-\left(\mathrm{EFFECTIVE}\;\mathrm{PATIENT}\;\mathrm{DIAMETER}-303\right)\times0.008678)$$

This differs from size-specific dose estimate, which normalizes to obtain consistent dose per unit of tissue as reflected in a phantom [23, 24]. Our size normalization mitigates the effect of patient size on dose, using the relationship between size and dose observed in our dataset. The goal of our approach—in the context of comparing dose across protocols and hospitals—is to ensure the impact of patient size on doses is eliminated.

### Patient size

Patients were divided by size in several ways to analyze the variation in radiation dose and technical parameters. First, patients were divided into deciles based on patient diameter in millimeters [21]. Second, because deciles may be too crude to measure changes in technical parameters that occur with change in patient diameter, as a second approach, patients were double-stratified into three size categories based on patient diameter, defined as small if they were less than or equal to the 25^th^ percentile, medium if between 25 and 75^th^ percentiles, and large if above the 75^th^ percentile. Then within each size category, patients were divided into deciles (= 30 deciles).

### Characterization of imaging protocols

We identified each protocol in the registry used for routine abdomen and pelvis indications (including all scans of the abdomen, pelvis, or combined abdomen and pelvis), defining a protocol as a unique combination of protocol name and specific scanner used. We determined whether each protocol was used in one, two, or all three patient size categories. To qualify, at least 20 patients in a size category had to have been imaged with a given protocol.

For each protocol, we used medians to summarize the radiation dose metrics and technical parameters of its constituent CT exams, including those directly reported by the scanner (CTDI_vol_, DLP, kV, mAs, collimation, pitch, and scan length) and those calculated (patient diameter, size-adjusted CTDI_vol_, size-adjusted DLP, effective dose, effective mAs, and number of phases). The scan length was defined as the total irradiated region and for multiple-phase studies was the average across irradiating events. For each exam, when there were multiple irradiating events, the DLP, size-adjusted DLP, ED, and number of phases were summed across the irradiating events, whereas the CTDI_vol_, size-adjusted CTDI_vol_, mAs, effective mAs, scan length, and pitch were averaged and weighted by scan length across irradiating events. Bolus scans were not included.

### Statistical analysis

CT scans missing dose metrics or technical parameters were not included. In order to exclude outliers, we dropped scans with values less than the 0.1^th^ percentile. Because scanner model may contribute to dose, we included only CT scans obtained on scanner models where at least 5 individual scanners of that model exist in the registry.

Each CT scan was assigned to one of the three patient size categories (small, medium, large). Descriptive statistics were examined for demographic variables, radiation dose metrics, and the technical parameters as they varied by patient size. Stratifying by decile of patient size, we calculated mean (and 95% CI calculated using bootstrapping) percent increase in radiation dose metrics and technical parameters per decile increase in size, and we calculated the percent increase (and 95% CI using bootstrapping) of the dose metrics and technical parameters in the highest compared with lowest deciles. We then repeated these analyses for each of the three patient size categories.

Protocols were next stratified by decile of size-adjusted DLP to illustrate the variation in dose. For each protocol, we calculated the median size-adjusted DLP, and then sorted and grouped into deciles of median size-adjusted DLP. Within each decile of size-adjusted DLP, we calculated the mean values (and 95% CI) of dose and technical parameters for the constituent protocols, and—to quantify variation in dose and parameters—we calculated the mean percent change (and 95% CI using bootstrapping) for each variable per decile increase in size-adjusted DLP and between the 1^st^ (lowest dose) to 10^th^ (highest dose) deciles. We then repeated these analyses for each of the three patient size categories.

To identify which technical parameters contributed the most to observed between-protocols variation in dose, we combined the observed percent increases between deciles of the technical parameters with established understanding of parameter-dose relationship in medical physics. Specifically, for an exam in which a single technical parameter sees an *X*% increase with all other factors kept constant, the DLP will see the same *X*% increase for effective mAs, scan length, or number of phases, whereas the DLP will see an even greater increase [(1 + *X*)^2.5^–1]% for an *X*% increase in kV [25]. Using these established equations, for each technical parameter, we computed the expected increase in size-adjusted DLP per decile due to the observed change in the parameter. Technical parameters that contribute strongly to between-protocols dose variation should induce an expected DLP increase per decile similar to the observed size-adjusted DLP increase per decile.

Finally, to estimate the potential impact of widespread adoption of optimized protocols for routine abdomen CT, we first calculated the total dose to all patients in this study by summing the size-adjusted doses for all examinations. We then recalculated the total dose under two scenarios. First, we defined the target routine abdomen protocol dose as 433 mGy-cm. This is the median (achievable dose (AD)) for acute appendicitis in Europe using the EUCLID framework [26]. We then (1) summed all observed doses for patients who were scanned with protocols with a mean at or below 433 (their doses do not change), (2) summed all observed doses for remaining patients who were scanned with size-adjusted DLP at or below 433 (their doses also do not change), and (3) multiplied the remaining patients by 433 mGy-cm (to reflect their new optimized doses in this hypothetical calculation), and (4) summed the values produced by (1), (2), and (3) to produce a “post-optimization population total dose.” Second, we repeated this calculation using a target dose of 645 mGy-cm, which reflects the median dose of acute appendicitis in the USA using the EUCLID framework [26]. This value is similar to the median dose for abdomen CT reported by the American College of Radiology (AD = 615 mGy-cm for abdomen and pelvis with contrast and AD = 657 mGy-cm for abdomen and pelvis without contrast) [15]. We then estimated the total dose saved by computing the percent reduction between the observed and hypothetical post-optimization population total doses in both scenarios.

## Results

A total of 748,846 routine abdomen CT scans were included (Table 1). There were more CT scans obtained in women (*n* = 426,840, 57%) and the number of scans increased with age and peaked in those aged 60–69 years (*n* = 142,551, 19%). Most scans were obtained on GE (*n* = 298,316, 39.8%) or Siemens (*n* = 244,778, 32.7%) scanners, and most were performed in the USA (*n* = 665,301, 88.8%).

The data included 1033 protocols collected from 95 facilities and 242 scanners. The mean number of scans per protocol was 725 (median = 159, range = 20–22, 733). About half of all protocols (*n* = 547, 53%) were not used in a size-specific fashion, meaning they were used for patients across all three size categories (Supplemental Table 1). Of protocols used selectively, 225 (21.8%) were used in patients from two size categories, while 261 (25.3%) were used in patients from a single category: 23 (2.2%) were used only in small patients; 172 (16.7%) in medium patients; and 66 (6.4%) in large patients.

### Variation in radiation dose and technical parameters by patient size category

The mean values for patient diameter, radiation dose metrics, and technical parameters overall, and stratified by the three size categories, are provided in Table 2. As expected, the unadjusted CTDI_vol_ and DLP values increased across the categories, more than doubling between small and large. Effective dose also increased steadily between categories. By contrast, size-adjusted CTDI_vol_ and size-adjusted DLP did not change meaningfully by size category, demonstrating that the radiation dose variation attributed to patient size was eliminated through size adjustment.

**Table 2:** Radiation dose metrics and technical parameters overall and stratified by patient size category

Variable (units)	Patient size category							
	All patients		Small		Medium		Large	
	*n* = 748,846		*n* = 165,816		*n* = 360,850		*n* = 222,180	
	Mean	Std Dev	Mean	Std Dev	Mean	Std Dev	Mean	Std Dev
Patient diameter (mm)	309	(45)	252	(17)	301	(16)	363	(28)
CTDI_vol_ (mGy)	13	(7.2)	8	(3.3)	12	(4.9)	20	(7.8)
DLP (mGy-cm)	798	(546)	447	(283)	701	(404)	1217	(629)
Effective dose (mSv)	13	(8)	9	(6)	12	(7)	16	(9)
mAs	181	(89)	120	(55)	166	(68)	251	(94)
Pitch^*^	1.0	(0.3)	1.0	(0.2)	1.0	(0.3)	1.0	(0.3)
Effective mAs	183	(95)	118	(54)	166	(67)	260	(106)
kV (kilovoltage)	119	(7.5)	116	(8.0)	118	(7.4)	121	(6.8)
Scan length (cm)	48	(9.2)	45	(8.8)	48	(9.0)	51	(9.2)
Slice thickness (mm)	3.7	(1.6)	3.7	(1.7)	3.7	(1.6)	3.7	(1.6)
Number of phases	1.3	(0.7)	1.3	(0.7)	1.3	(0.7)	1.3	(0.6)
Calculated size-adjusted variables								
Size-adjusted CTDI_vol_ (mGy)	12	(4.6)	11	(5.0)	12	(4.6)	12	(4.2)
Size-adjusted DLP (mGy-cm)	702	(389)	690	(448)	701	(388)	712	(338)

*Abbreviations*: *CTDI*_*vol*_ volumetric computed tomography dose index, *DLP* dose length product, *mAs* milliampere-seconds, *mGy* milliGray, *mSv* millisieverts

^*^Table distance traveled in one 360° gantry rotation divided by beam collimation

Some technical parameters also increased across the size categories: e.g., mAs and effective mAs were approximately twice the value in large as small patients, which suggests automatic exposure control (AEC) may have been used. Notably, average kV and scan length were virtually unchanged with size category, while the number of phases as expected did not change (Table 2).

### Variation in radiation dose and technical parameters by patient size deciles

**Fig. 1:**
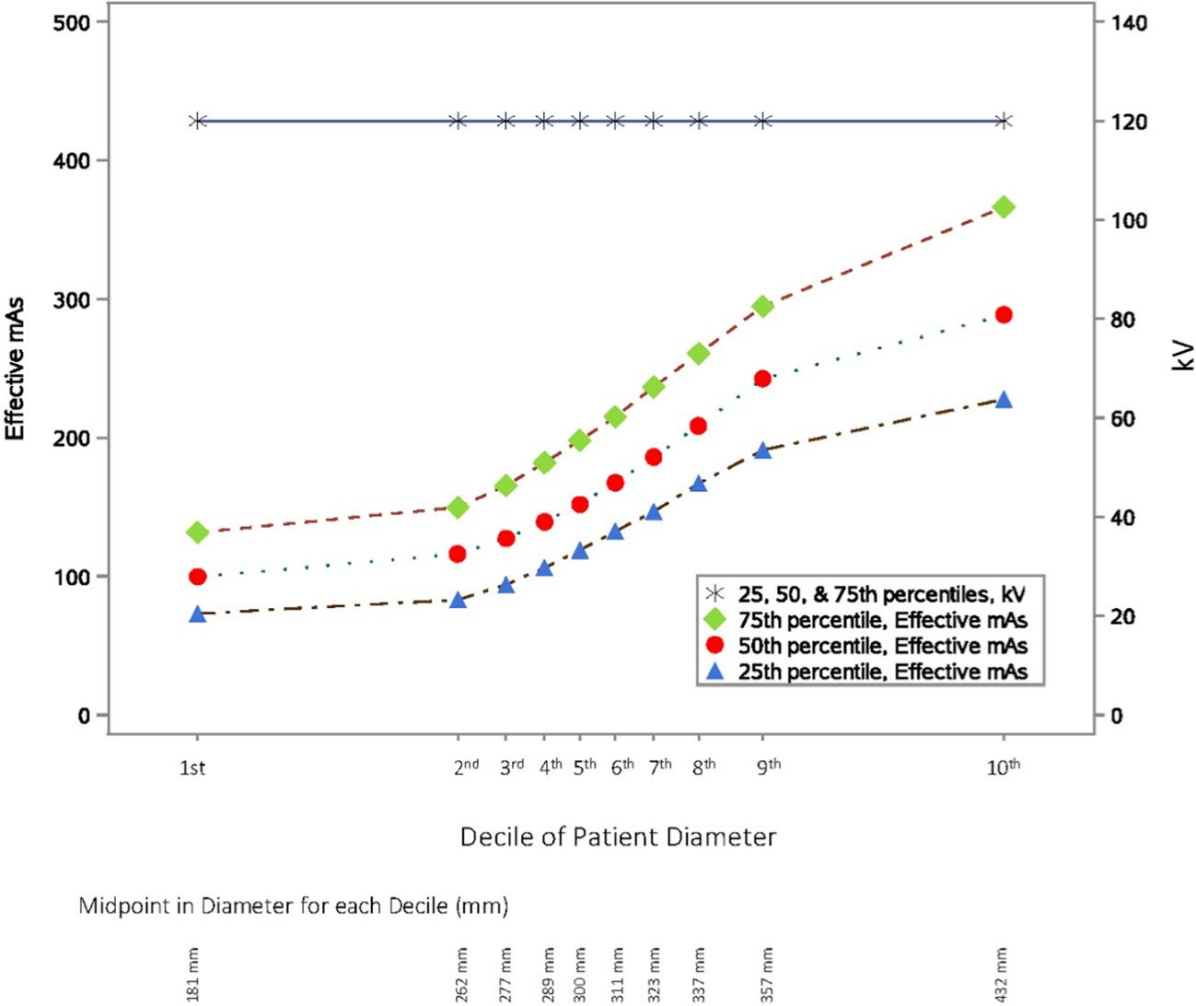
Effective mAs and kV (25^th^, 50^th^, and 75^th^ percentiles in distribution) by decile in effective patient diameter

Mean dose metrics and technical parameters by decile of patient diameter are shown in Table 3. Each decile includes between 74,883 and 74,888 CT scans. Average patient diameter ranged from 238 mm for patients in the first decile to 395 mm in the highest decile, reflecting an average 6% increase per decile (Table 3). Again, we observed the expected increase in unadjusted CTDI_vol_ and DLP by size, reflecting an average increase of 15% and 16% per decile, respectively (0.2% and –0.54% per decile in the corresponding size-adjusted dose metrics). Similarly, effective mAs increased with patient size (12% per decile), yet again there was minimal (1%) change per decile in kV (116 in the 1^st^ compared to 122 in the 10^th^ reflecting an average percent increase per decile = 0.54% [95% CI 0.53–0.54%]). Figure 1 show the distribution of effective mAs and kV by patient decile, demonstrating a consistent increase in effective mAs with patient diameter but no change in kV.

**Table 3:** Radiation dose metrics and technical parameters for CTs sorted by decile in patient size based on effective patient diameter. Each decile includes between 74,883 and 74,888 patients and reflects the mean for all constituent patients. The mean percent increase per decile increase in effective patient diameter, and the percent increase between the 1^st^ and 10^th^ deciles of diameter, is shown for all variables with the 95% confidence interval. Because these reflect mean values, the results may reflect a number that is implausible for an individual scan (e.g., kV = 116). For the percent change, the numbers are rounded to the nearest 1% unless estimate < 10%, where numbers show with 2 significant digits

Decile abdomen diameter		Patient diameter (mm)	CTDI_vol_ (mGy)	Size-adjusted CTDI_vol_ (mGy)	DLP (mGy-cm)	Size-adjusted DLP (mGy-cm)	Effective Dose (mSv)	Pitch^*^	Effective mAs	Kilovoltage (kV)	Scan length (cm)	Number of phases
		Mean (95% CI)	Mean (95% CI)	Mean (95% CI)	Mean (95% CI)	Mean (95% CI)	Mean (95% CI)	Mean (95% CI)	Mean (95% CI)	Mean (95% CI)	Mean (95% CI)	Mean (95% CI)
	1st	238 (238,238)	7 (7,7)	12 (12,12)	403 (401,405)	709 (705,712)	9 (9,9)	1.04 (1.03,1.04)	109 (109,110)	116 (116,116)	45 (45,45)	1.4 (1.3,1.4)
	2nd	262 (262,262)	8 (8,8)	11 (11, 11)	475 (473, 477)	675 (672, 678)	9 (9,10)	1.03 (1.03, 1.03)	124 (124, 124)	117 (117, 117)	46 (46,46)	1.3 (1.3, 1.3)
	3rd	277 (277,277)	9 (9,9)	11 (11,11)	539 (537, 541)	675 (672, 678)	10 (9,10)	1.03 (1.03, 1.03)	137 (136, 137)	117 (117, 117)	47 (47,47)	1.3 (1.3, 1.3)
	4th	289 (289,289)	10 (10,10)	12 (12, 12)	608 (606, 611)	687 (684, 690)	11 (11,11)	1.02 (1.02, 1.03)	149 (149, 150)	118 (118, 118)	47 (47,47)	1.3 (1.3, 1.3)
	5th	300 (300,300)	11 (11,12)	12 (12, 12)	682 (679, 685)	699 (696, 702)	12 (12,12)	1.02 (1.02, 1.02)	163 (163, 164)	118 (118, 118)	48 (48,48)	1.3 (1.3, 1.3)
	6th	311 (311,311)	13 (13,13)	12 (12, 12)	764 (762, 767)	712 (709, 715)	12 (12,13)	1.02 (1.02, 1.02)	178 (177, 178)	119 (119, 119)	49 (49,49)	1.3 (1.3, 1.3)
	7th	323 (323,323)	14 (14,14)	12 (12, 12)	865 (862, 868)	727 (725, 730)	13 (13,13)	1.02 (1.02, 1.02)	195 (195, 196)	119 (119, 119)	49 (49,49)	1.3 (1.3, 1.3)
	8th	337 (337,337)	16 (16,16)	12 (12, 12)	989 (986, 993)	737 (734, 740)	14 (14,15)	1.01 (1.01, 1.01)	217 (217, 218)	120 (120, 120)	50 (50,50)	1.3 (1.3, 1.3)
	9th	356 (356,356)	19 (19,19)	12 (12, 12)	1152 (1148, 1156)	728 (726, 731)	16 (16,16)	1.01 (1.00, 1.01)	248 (247, 249)	120 (120, 120)	51 (51,51)	1.3 (1.3, 1.3)
	10th	395 (395,395)	24 (24,24)	12 (12, 12)	1500 (1495, 1505)	672 (669, 674)	19 (19,19)	0.99 (0.99, 0.99)	312 (311, 313)	122 (122, 122)	52 (52,52)	1.3 (1.3, 1.3)
Percent change 1^st^ to 10^th^ deciles (95% CI)^**^		66% (66%, 66%)	241% (239%, 242%)	1.5%(1.1%, 1.9%)	272% (270%, 274%)	-5.3% (-5.8% -4.7%)	126% (124%, 127%)	-4.3% (-4.5%, -4.0%)	186% (185%, 187%)	4.9% (4.9%, 5.0%)	17%(17%, 17%)	-4.7% (-5.2%, -4.3%)
Mean percent increase per decile (95% CI)^**^		5.8% (5.8%, 5.8%)	15% (15%,1 5%)	0.20% (0.15%, 0.24%)	16% (16%, 16%)	-0.54% (-0.60%, -0.48%)	9.6% (9.5%, 9.7%)	-0.48% (-0.51%, -0.45%)	12% (12%, 13%)	0.54% (0.53%, 0.54%)	1.7% (1.7%, 1.8%)	-0.53% (-0.59%, -0.48

*Abbreviations*: *CI *confidence interval, *CTDI*_*vol*_ volumetric computed tomography dose index, *DLP* dose length product, *mAs* milliampere-seconds, *mGy* milliGray, *mSv* millisieverts

^*^Table distance traveled in one 360° gantry rotation divided by beam collimation

^**^95% bootstrap confidence intervals using 1,000 bootstrap samples of size 748,846

We replicated this analysis separately by deciles within each of the three size categories with similar results (Table 4 and Supplemental Table 2). Across the categories, the average kV was 116 in the 1^st^ decile of small patients (average diameter = 217 mm) and 123 in the 10^th^ decile of large patients (average diameter = 423 mm). The average change in kV was 0.1–0.3% between deciles. This contrasts with effective mAs where the average change in effective mAs between deciles was 20-fold higher, 3.0–6.0% per decile. The magnitude of the changes in the other radiation dose metrics and technical parameters was greatest among large patients (Table 4). For example, unadjusted CTDI_vol_ and DLP both increased by 4% per decile in small patients and 7% in large patients (Table 4).

**Table 4:** The average percent increase in patient diameter, dose metrics, and technical parameters per decile increase in patient size within each of the three size categories (small, medium, large). Results shown to the nearest 0.1%

	Patient Diameter (mm)	CTDI_vol_ (mGy)	Size-adjusted CTDI_vol_ (mGy)	DLP (mGy-cm)	Size-adjusted DLP (mGy-cm)	Effective dose (mSv)	Effective mAs	Kilovoltage (kV)	Scan length (cm)
All Patients	5.8%	14.7%	0.2%	15.8%	-0.5%	9.6%	12.5%	0.5%	1.7%
Patients by size category									
Small	2.5%	3.7%	-1.1%	3.8%	-1.5%	2.9%	3.0%	0.1%	1.0%
Medium	1.9%	5.2%	0.8%	5.7%	0.8%	3.1%	4.2%	0.2%	0.6%
Large	2.6%	6.6%	-1.0%	7.1%	-1.6%	5.3%	6.0%	0.3%	0.7%

*Abbreviations*: *CTDI*_*vol*_ volumetric computed tomography dose index, *DLP* dose length product, *mAs* milliampere-seconds

### Variation in technical parameters by radiation dose deciles

The 1033 unique protocols were next sorted by median size-adjusted DLP, and the average (mean) values of technical parameters and radiation dose metrics for each decile in size-adjusted DLP are shown in Table 5. The mean size-adjusted DLP increased 535% between the lowest and highest deciles. This difference was not driven by patient size, as diameter changed little across the deciles (range 309–321 mm). Rather, high size-adjusted DLPs resulted from acquisition techniques, most notably effective mAs and the number of phases. The effective mAs increased from 125 to 266 from the lowest to highest decile (113% increase), and the average number of phases more than doubled from 1.1 to 2.6 across deciles (149% increase). The fact that size-adjusted CTDI_vol_ increased considerably across the deciles (166%), but nowhere close to the rate of adjusted DLP (535%), suggests that phase number is a strong driver of the change in DLP.

**Table 5:** Routine abdomen and pelvis CT protocols, sorted by size-adjusted dose length product (DLP). There are 103 or 104 protocols in each decile, and there are a mean of 288–1381 CTs per protocol. For each decile, the mean radiation dose metrics and values for the technical parameters are provided. The mean percent change in each technical parameter and radiation dose metric for decile increase in size-adjusted DLP is provided, as is the percent change from the 1^st^ to the 10^th^ deciles in size-adjusted DLP. Lastly, the expected increase in DLP per decile due to observed variation in parameter (effective mAs, kV, scan length, and number of phases) is provided to quantify which protocols are the most important contributors to the observed variation in size-adjusted DLP

Size-adjusted DLP decile		Patient diameter (mm)	DLP (mGy-cm)	Size-adjusted DLP (mGy-cm)	CTDI_vol_ (mGy)	Size-adjusted CTDI_vol_ (mGy)	Effective dose (mSv)	Effective mAs	Kilovoltage (kV)	Scan length (cm)	Number of phases
		Mean (95% CI)	Mean (95% CI)	Mean (95% CI)	Mean (95% CI)	Mean (95% CI)	Mean (95% CI)	Mean (95% CI)	Mean (95% CI)	Mean (95% CI)	Mean (95% CI)
	1st	309 (302,315)	295 (275,315)	265 (254,275)	7 (6,7)	6 (6,7)	4 (4,5)	125 (114,135)	115 (113,117)	42 (39,44)	1.1 (1.0,1.1)
	2nd	306 (302,310)	387 (369,405)	366 (361,370)	8 (8,9)	8 (7,8)	6 (6,6)	141 (131,151)	116 (114,118)	47 (45,49)	1.0 (1.0,1.1)
	3rd	309 (305,313)	477 (458,497)	441 (437,445)	10 (9,10)	9 (9,10)	7 (7,7)	167 (153,181)	117 (115,118)	48 (46,49)	1.0 (1.0,1.0)
	4th	306 (303,310)	518 (499,538)	496 (492,499)	10 (10,11)	10 (10,10)	8 (8,9)	164 (151,177)	117 (115,118)	48 (46,49)	1.1 (1.0,1.2)
	5th	308 (304,312)	614 (588,640)	563 (560,567)	12 (12,13)	12 (11,12)	10 (9,10)	185 (172,197)	119 (117,120)	47 (45,48)	1.1 (1.0,1.2)
	6th	309 (304,314)	695 (657,732)	630 (627,634)	13 (12,14)	12 (12,13)	11 (11,11)	181 (170,192)	118 (117,120)	46 (45,48)	1.2 (1.1,1.3)
	7th	313 (308,319)	825 (772,877)	712 (707,717)	15 (14,16)	13 (13,14)	13 (12,13)	209 (194,224)	119 (117,120)	45 (43,47)	1.4 (1.3,1.5)
	8th	312 (307,317)	943 (886,1000)	818 (810,826)	16 (15,18)	15 (14,15)	15 (14,15)	214 (199,230)	120 (119,121)	45 (44,47)	1.4 (1.3,1.6)
	9th	321 (312,330)	1297 (1188,1405)	1015 (1001,1030)	20 (17,22)	15 (14,16)	19 (18,20)	296 (252,340)	121 (119,122)	47 (45,49)	1.7 (1.6,1.9)
	10th	312 (304,320)	1892 (1753,2032)	1680 (1576,1785)	20 (18,23)	17 (16,19)	30 (28,32)	266 (229,303)	119 (119,120)	44 (42,45)	2.6 (2.4,2.8)
Percent change 1^st^ to 10^th^ decile (95% CI)^1^		1.0% (-0.4%, 1.7%)	541% (513%, 562%)	535% (531%, 547%)	194% (174%, 205%)	166% (160%,176%)	592% (561%, 610%)	113% (96%, 118%)	4.0% (3.7%, 5.0%)	4.6% (1.6%, 5.6%)	149% (141%, 154%)
Mean percent increase per decile (95% CI%)^1^		0.1% (0.0%, 0.2%)	23% (23%, 24%)	24% (24%, 24%)	13% (12%, 13%)	12% (11%, 12%)	25% (24%, 25%)	9.6% (8.5%, 9.7%)	0.4% (0.4%, 0.6%)	0.6% (0.3%, 0.8%)	12% (11%, 12%)
Expected increase in DLP per decile due to observed variation in parameter								10%	1%	1%	12%

*Abbreviations*: *CTDI*_*vol*_ volumetric computed tomography dose index, *CI* confidence interval, *DLP* dose length product, *kV* kilovoltage, *mAs* milliampere-seconds, *mGy* milliGray, *mSv* millisieverts

^**1**^95% bootstrap confidence intervals using 1000 bootstrap samples of size 748,846

The contribution of each technical parameter to the observed 24% increase in size-adjusted DLP per decile is also shown in Table 5. For kV, the observed 0.4% increase in kV per decile is expected to result in only a small (1%) increase in size-adjusted dose. Because this is far smaller than the observed 24% change in size-adjusted dose, it suggests that kV was not a large contributor to between-protocols variation. Conversely, the larger observed increase in effective mAs (10%) and number of phases (12%) suggests these to be greater contributors to between-protocols dose variation, with number of phases being the most contributory.

This analysis was repeated, stratified within size category, producing similar results (Supplemental Table 3), with the range of size-adjusted DLP remarkably similar for all three size categories. For small patients, the size-adjusted DLP ranged from 259 to 1546 mGy-cm; medium patients 258–1705; and large patients 294–1549.

### Impact of population dose optimization

The broad adoption of optimized protocol thresholds would result in population dose reductions of 40.0% (using a target of 433 mGy-cm) or 18.6% (using 645 mGy-cm) from current practice.

## Discussion

We have described the technical parameters and dose metrics for almost 750,000 routine abdomen CT scans across diverse radiology practices. These show tremendous variation in radiation dose (535% in size-adjusted DLP) that is not driven by patient size but rather by how patients are scanned, particularly by manipulation of effective mAs and phase. The results also demonstrate that while automatic exposure control (AEC) is widely used to modify mAs, kV is almost never adjusted despite this being a demonstrated best practice [19, 27, 28]. Unindicated scan phases are the leading source of unnecessarily high radiation doses [29] and our results demonstrate high correlation of phase number with dose increases; protocols with the lowest size-adjusted doses used one scan phase, whereas those in the highest decile used an average of 2.6.

There are several approaches for dose optimization. One method is to develop protocols tailored to patient size, as it may be difficult for a single protocol to work across a large range of sizes even when AEC is available [18–20]. In addition to adjusting mAs and kV, a well-optimized protocol would also adjust scan speed allowing dose adjustment for size. For example, if moving from a larger to a smaller patient protocol, in addition to using a lower kV and less mA, the helical pitch might increase and the tube rotation time decrease, which increases scan speed while lowering the maximum dose delivered [20]. However, we did not observe a systematic pattern of protocols being used in a size-specific manner, with only a quarter used in one size category. If this reflects a belief that multiple protocols are an administrative burden, approaches for simplified protocol management exist to ease this obstacle [30].

A second strategy is to tailor kV by patient size [31], which would lower doses particularly among smaller patients. Manufacturers have offered some scanners in recent years that make it easy to modify kV (such as Siemens’ Care KV, Canon’s SURE kV, and GE’s kV assist), but manual manipulation is and always has been available on all scanners. Yet we found kV is virtually never changed, even on machines that can automate kV selection. Reducing kV not only would reduce dose, but can also provide image quality advantages, such as improving contrast conspicuity in CT angiography, improved assessment of mural hyperenhancement in Crohn’s disease, and reducing contrast volume in a patient with chronic kidney disease. A third approach is to reduce unnecessary scan phases. While routine abdomen scans should be performed using a single-phase approach [21], we observed strong variation in number of phases, and more than half of scans used more than one phase.

The hypothetical dose optimization analyses demonstrate the magnitude of dose reduction that could be achieved (18.6–40.0%) if more practices adopted the optimized protocols widely used at some facilities. The target dose of 433 mGy-cm reflects a protocol around the 3^rd^ decile in our distribution of size-adjusted DLP (where the mean effective mAs is 167, CTDI_vol_ is 10 mGy) whereas the target dose of 645 mGy-cm reflects a target protocol around the 6^th^ decile (mean effective mAs is 181 and CTDI_vol_ 13 mGy).

The strengths of this study are its large sample size, inclusion of diverse practices, and detailed technical parameter data. There are several limitations. First, we classified scans as being done for routine abdomen indications based on the study description and protocol name in the DICOM data, and we have shown this approach to be 90% accurate compared to expert chart review [21]. Nonetheless, some scans will have been misclassified as routine, but this is unlikely to impact the primary conclusions. Second, we calculated size-adjusted DLP based only on patient diameter and did not adjust for patient height, and taller patients may be represented more among those who received higher dose examinations. However, this is expected to have contributed only minimally to the larger variation in dose, as scan length changed only modestly across the observed deciles in dose. Third, we have only focused on a single indication for CT (albeit the most common indication); future studies should explore other reasons for imaging, such as oncologic care. Lastly, we did not assess image quality in this study, and thus cannot assess the impact of the observed dose variation on radiologists’ satisfaction with quality. However, all scans were obtained for routine care and were deemed adequate for diagnosis when they were obtained.

This work shows, in the actual practice of almost 100 imaging facilities, a large variation in radiation doses exceeding that required by patient size or scanning indication, as well as widespread failure to implement best practices beyond use of AEC. Broader adoption of evidence-based practices including using size-specific protocols, increasing manipulation of kV, and eliminating multiphase protocols would reduce this variation and improve the safety of routine abdominal imaging.

### Supplementary information

Below is the link to the electronic supplementary material.

Supplementary file1 (PDF 157 KB)

## Abbreviations

AEC: Automatic exposure control
CTDI_vol_: Volume computed tomography dose index
DICOM: Digital Imaging and Communications in Medicine
DLP: Dose length product
kV: Kilovolt
mAs: Milliampere seconds
mGy: Milligray

## Declarations

### Guarantor

The scientific guarantor of this publication is Dr. Rebecca Smith-Bindman.

### Statistics and biometry

Four biostatisticians included as coauthors on the manuscript contribute to the design and statistical analysis of the study including Dr. Kang, Mr. Chu, Dr. Wang, and Dr. Chung.

### Study subjects

The data derive from The UCSF International CT dose registry, and some of the patients described in this report may have also been included in prior publications including the following referenced publications.

Bos D, Yu S, Luong J et al (2021) Diagnostic reference levels and median doses for common clinical indications of CT: findings from an international registry. Eur Radiol. https://doi.org/10.1007/s00330-021-08266-1

Smith-Bindman R, Wang Y, Chu P et al (2019) International variation in radiation dose for computed tomography examinations: prospective cohort study. BMJ 364:k4931

Smith-Bindman R, Yu S, Wang Y et al (2022) An image quality-informed framework for CT Characterization. Radiology 302:380-389

Chu PW, Yu S, Wang Y et al (2021) Reference phantom selection in pediatric computed tomography using data from a large, multicenter registry. Pediatr Radiol. https://doi.org/10.1007/s00247-021-05227-0

However, the aim of this work is to understand specific quality practices for routine abdomen CT and is unrelated to previous studies. There is no overlap in the methodology.

### Methodology

• Prospective

• Observational

• Multicenter study
