# Supplementary material for: Large variation in radiation dose for routine abdomen CT: reasons for excess and easy tips for reduction
Source: Eur Radiol. 2023 Sep 21;34(4):2394–404. doi: 10.1007/s00330-023-10076-6 (PMC10957641; doi:10.1007/s00330-023-10076-6)
Supplement: Supplementary file 1 — Supplementary file1 (PDF 157 KB) [file 330_2023_10076_MOESM1_ESM.pdf]

Large variation in radiation dose for routine abdomen CT: reasons for excess and easy tips for reduction

Supplemental Tables

Supplemental Table 1: For each of the 1033 routine abdomen protocols (x-axis) the percent of patients imaged with that protocol from each of the three size categories.

	All Patients N = 748,846		Used in Specific Patient Size Groups		
			Small N= 165,816	Medium N=360,850	Large N=222,180
	Number	Percent	N (%)	N (%)	N (%)
Total number of protocols	1033				
Protocols used in one size category	261	(25.3%)	23 (2.2%)	172 (16.7%)	66 (6.4%)
Protocols used in small and medium size patients	58	(5.6%)	-------------------	-------------------	
Protocols used in medium and large size patients	167	(16.2%)		-------------------	-------------------
Protocols used in three size categories	547	(53.0%)	-------------------	-------------------	-------------------

Supplemental Table 2: Technical parameters and radiation dose metrics within each of the three size categories, sorted by decile in patient diameter within each of the size categories. Shown for 1a) small patients 1b) medium patients 1c) large patients. Slice thickness shows the mode, the remaining variables show the mean.

Supplemental Table 2a: Small patients

Decile in Abdominal Diameter	N	Patient Diameter (mm)	$CTDI_{vol}$ (mGy)	DLP (mGy-cm)	ED (mSv)	Pitch	Effective mAs	kV (kilovoltage)	Scan Length (cm)	Slice Thickness (mm)	N Phases
1st	16,581	217	6	369	8	1.03	102	116	43	5	1.4
2nd	16,581	235	7	392	8	1.04	106	116	45	5	1.4
3rd	16,582	243	7	408	9	1.04	110	116	45	5	1.3
4th	16,580	249	7	423	9	1.04	114	116	45	5	1.3
5th	16,582	253	8	447	9	1.04	118	116	46	5	1.3
6th	16,580	258	8	456	9	1.03	120	117	46	5	1.3
7th	16,584	262	8	469	9	1.03	123	117	46	5	1.3
8th	16,582	265	8	486	10	1.03	126	117	46	5	1.3
9th	16,581	268	9	501	10	1.03	129	117	46	5	1.3
10th	16,583	272	9	516	10	1.03	133	117	46	5	1.3
Increase per decile		3%	4%	4%	3%	0%	3%	0%	1%	0%	-1%
Relative 10th vs 1st decile		1.3	1.4	1.4	1.3	1.0	1.3	1.0	1.1	1.0	0.9

Abbreviations: $CTDI_{vol}$ = volumetric computed tomography dose index, DLP= Dose-Length Product, mAs = milliampere-seconds, mGy = milliGray, mSv = millisieverts

Supplemental Table 2b, Average patients

Decile in Abdominal Diameter	N	Patient Diameter (mm)	$CTDI_{vol}$ (mGy)	DLP (mGy-cm)	ED (mSv)	Pitch	Effective mAs	kV (kilovoltage)	Scan Length (cm)	Slice Thickness (mm)	N Phases
1st	36,083	276	9	535	10	1.03	136	117	47	5	1.3
2nd	36,083	282	10	570	10	1.03	142	117	47	5	1.3
3rd	36,086	288	10	602	11	1.03	148	118	47	5	1.3
4th	36,087	293	11	636	11	1.02	154	118	48	5	1.3
5th	36,086	299	11	673	12	1.02	161	118	48	5	1.3
6th	36,084	304	12	710	12	1.02	169	118	48	5	1.3
7th	36,079	309	13	751	12	1.02	175	118	48	5	1.3
8th	36,091	315	13	793	13	1.02	183	119	49	5	1.3
9th	36,077	320	14	845	13	1.02	192	119	49	5	1.3
10th	36,094	326	15	897	14	1.02	200	119	49	5	1.3
Increase per decile		2%	5%	6%	3%	0%	4%	0%	1%	0%	0%
Relative 10th vs 1st decile		1.2	1.6	1.7	1.3	1.0	1.5	1.0	1.1	1.0	1.0

Abbreviations: $CTDI_{vol}$ = volumetric computed tomography dose index, DLP= Dose-Length Product, mAs = milliampere-seconds, mGy = milliGray, mSv = millisieverts

Supplemental Table 2c, Large patients

Decile in Abdominal Diameter	N	Patient Diameter (mm)	$CTDI_{vol}$ (mGy)	DLP (mGy-cm)	ED (mSv)	Pitch	Effective mAs	kV (kilovoltage)	Scan Length (cm)	Slice Thickness (mm)	N Phases
1st	22,214	332	15	943	14	1.01	209	119	50	5	1.3
2nd	22,222	336	16	986	14	1.02	216	120	50	5	1.3
3rd	22,217	341	17	1027	15	1.01	224	120	50	5	1.3
4th	22,217	346	17	1073	15	1.01	232	120	50	5	1.3
5th	22,218	352	18	1129	15	1.01	243	120	51	5	1.3
6th	22,219	359	19	1180	16	1.00	254	120	51	5	1.3
7th	22,218	367	20	1254	16	1.00	267	121	51	5	1.3
8th	22,218	378	22	1344	17	0.99	284	121	52	5	1.3
9th	22,219	393	24	1473	19	0.99	310	122	52	5	1.3
10th	22,218	423	28	1762	23	0.99	355	123	53	5	1.3
Increase per decile		3%	7%	7%	5%	0%	6%	0%	1%	0%	0%
Relative 10th vs 1st decile		1.3	1.8	1.9	1.6	1.0	1.7	1.0	1.1	1.0	1.0

Abbreviations: $CTDI_{vol}$ = volumetric computed tomography dose index, DLP= Dose-Length Product, mAs = milliampere-seconds, mGy = milliGray, mSv = millisieverts

Supplemental Table 3: Protocols sorted by decile of size-adjusted DLP showing the average technical parameters for each decile within each of the three size categories. Shown for 1a) small patients 1b) medium patients 1c) large patients. Slice thickness shows the mode, the remaining variables show the mean.

Supplemental Table 3a, Small patients

Decile in Size-Adjusted DLP	Number of Protocols	Average CTs per protocol	Patient Diameter (mm)	$CTDI_{vol}$ (mGy)	Size -Adjusted DLP (mGy-cm)	ED (mSv)	Effective mAs	Scan Length (cm)	kV (kilovoltage)	N Phases
1st	62	171	253	4	259	3	70	45	109	1
2nd	63	160	256	5	342	4	94	47	114	1
3rd	63	157	256	6	409	5	97	47	116	1.1
4th	63	274	255	6	465	6	104	48	116	1
5th	63	348	256	7	521	7	105	47	117	1
6th	62	307	255	8	573	7	125	45	117	1.1
7th	63	374	256	9	636	8	131	44	119	1.1
8th	63	397	257	9	739	10	126	43	117	1.5
9th	63	235	255	11	934	12	155	43	120	1.6
10th	63	217	253	11	1546	20	161	42	119	2.4
		Increase per decile	0%	13%	23%	24%	10%	-1%	1%	11%
		Relative 10th vs 1st Decile	1.0	3.0	6.0	6.3	2.3	0.9	1.1	2.3

Abbreviations: $CTDI_{vol}$ = volumetric computed tomography dose index, DLP= Dose-Length Product, mAs = milliampere-seconds, mGy = milliGray, mSv = millisieverts

Supplemental Table 3b, Medium patients

Decile in Size-Adjusted DLP	Number of Protocols	Average CTs per protocol	Patient Diameter (mm)	$CTDI_{vol}$ (mGy)	Size - Adjusted DLP (mGy-cm)	ED (mSv)	Effective mAs	Scan Length (cm)	kV (kilovoltage)	N Phases
1st	94	144	303	6	258	4	111	41	115	1.1
2nd	94	321	301	7	357	6	129	48	115	1.0
3rd	95	305	302	9	425	7	139	48	117	1.0
4th	94	283	302	10	475	7	157	47	116	1.1
5th	95	557	301	11	545	9	171	46	116	1.1
6th	94	790	302	12	620	10	176	47	118	1.2
7th	94	496	303	13	702	12	183	45	118	1.4
8th	95	397	302	14	812	14	188	45	120	1.5
9th	94	293	303	14	1002	17	197	45	120	1.9
10th	95	238	301	17	1705	29	209	42	120	2.7
		Increase per decile	2%	6%	14%	15%	4%	0%	0%	5%
		Relative 10th vs 1st Decile	1.0	2.7	6.6	7.2	1.9	1.0	1.0	2.5

Abbreviations: $CTDI_{vol}$ = volumetric computed tomography dose index, DLP= Dose-Length Product, mAs = milliampere-seconds, mGy = milliGray, mSv = millisieverts

Supplemental Table 3c, Large patients

Decile in Size-Adjusted Dose	Number of Protocols	Average CTs per protocol	Patient Diameter (mm)	$CTDI_{vol}$ (mGy)	Size-Adjusted DLP (mGy-cm)	ED (mSv)	Effective mAs	Scan Length (cm)	kV (kilovoltage)	N Phases
1st	78	122	357	10	294	6	183	45	119	1.1
2nd	78	245	356	14	416	9	236	50	119	1
3rd	78	220	356	16	492	11	244	50	119	1
4th	78	303	356	17	559	13	232	51	121	1.1
5th	78	536	357	19	619	14	251	51	120	1.2
6th	78	372	355	20	673	15	260	50	121	1.2
7th	78	262	359	22	732	17	286	50	120	1.2
8th	78	475	358	24	820	18	301	49	121	1.4
9th	78	172	366	28	999	23	392	52	120	1.4
10th	78	142	360	26	1549	35	337	47	120	2.5
		Increase per decile	2%	6%	11%	12%	6%	1%	0%	4%
		Relative 10th vs 1st Decile	1.0	2.5	5.3	5.8	1.8	1.0	1.0	2.2

Abbreviations: $CTDI_{vol}$ = volumetric computed tomography dose index, DLP= Dose-Length Product, mAs = milliampere-seconds, mGy = milliGray, mSv = millisieverts
